# Frequency of overweight and obesity in children and adolescents with autism and attention deficit/hyperactivity disorder

**DOI:** 10.1016/j.rppede.2015.12.006

**Published:** 2016

**Authors:** Arthur Kummer, Izabela Guimarães Barbosa, David Henrique Rodrigues, Natália Pessoa Rocha, Marianna da Silva Rafael, Larissa Pfeilsticker, Ana Cristina Simões e Silva, Antônio Lúcio Teixeira

**Affiliations:** a Faculdade de Medicina, Universidade Federal de Minas Gerais (UFMG), Belo Horizonte, MG, Brazil; b Universidade Federal de Juiz de Fora (UFJF), Governador Valadares, MG, Brazil

**Keywords:** Attention deficit and hyperactivity disorder, Austistic disorder, Pediatric obesity, Overweight

## Abstract

**Objective:**

To assess the frequency of overweight and obesity in children and adolescents with autism spectrum disorder (ASD) and with attention deficit/hyperactivity disorder (ADHD) and their parents, in comparison with children and adolescents without developmental disorders.

**Methods:**

Anthropometric measures were obtained in 69 outpatients with ASD (8.4±4.2 years old), 23 with ADHD (8.5±2.4) and 19 controls without developmental disorders (8.6±2.9) between August and November 2014. Parents of patients with ASD and ADHD also had their anthropometric parameters taken. Overweight was defined as a percentile ≥85; obesity as a percentile ≥95; and underweight as a percentile ≤5. For adults, overweight was defined as a BMI between 25 and 30kg/m^2^ and obesity as a BMI higher than 30kg/m^2^.

**Results:**

Children and adolescents with ASD and ADHD had higher BMI percentile (*p*<0.01) and *z*-score (*p*<0.01) than controls, and increased frequency of overweight and obesity (*p*=0.04). Patients with ASD and ADHD did not differ between them in these variables, nor regarding abdominal circumference. Parents of children with ASD and ADHD did not differ between themselves.

**Conclusions:**

Children and adolescents with ASD and ADHD are at a higher risk of overweight and obesity than children without developmental problems in the community.

## Introduction

Prevalence of overweight and obesity in developed countries is alarming and reaches 31.8% of children and adolescents.^1^ In the US, despite continued efforts to reduce these public health problems, rates have been stable in the last decade.^1^ In Brazil, epidemiological studies have shown an increased frequency of overweight and obesity in this age group.^2^^,^ 3 Comparison of the 1989 National Survey on Health and Nutrition (PNSN) and the 2008–2009 Consumer Expenditure Survey shows that overweight frequency in children between five and nine years old increased from about 9–33%.^3^

International studies have found an association between overweight/obesity and psychiatric disorders in children, such as autism spectrum disorder (ASD) and attention deficit/hyperactivity disorder (ADHD). Nevertheless, whether this association is characteristic of ASD and ADHD or common to behavioral and developmental problems in general remains unclear. Furthermore, the association is likely to be bidirectional; that is, not only behavioral problems may lead to obesity, but obesity may be a risk factor for the development of behavioral and developmental problems.^4^

Most studies of the nutritional status of young people and adults with ADHD reports a high frequency of overweight and obesity, as well as a mean body mass index (BMI) higher in patients with ADHD compared to controls without developmental disorders.^5^^–^ 7 The frequency of obesity is higher in adults with ADHD than in adults with childhood ADHD history, but whose symptoms remitted in adulthood.^8^ Similarly, obese young people also have higher frequency of ADHD.^5^ Furthermore, behavioral problems such as ADHD hinder obesity treatment.^9^

Similarly, studies also reported that children and adolescents with ASD are more often overweight and obese.^10^^–^ 13 In ASD, weight changes have been associated with sleep disorders,^10^^,^ 11 older age,^11^ and using food as a reward,^12^ among others. In addition, parents of children with autism are also more frequently obese.^14^ These factors suggest a complex interaction between genetic, molecular, and behavioral factors.

Despite these alarming data, there are no studies of the nutritional status of children with ASD and ADHD in Brazil. In addition, few studies have compared the BMI and/or the overweight frequency in different developmental disorders.

In this context, the aim of this study was to evaluate the frequency of overweight and obesity in children and adolescents with ASD and ADHD and their parents.

## Method

Children and adolescents seen in the outpatient clinics of ASD (n=69) and Attention Deficit (n=23) of the Psychiatry Service of the Hospital das Clinicas, Federal University of Minas Gerais (UFMG), Brazil, and their parents were invited to participate in this study. Data were collected between August and November 2014, and none of those invited declined to participate. All patients with ASD and ADHD met the diagnostic criteria of the DSM-5.^15^ It was considered the gold standard. Patients in ASD group had no ADHD as comorbidity and vice versa. Noteworthy, some patients with ASD were taking methylphenidate, but medical records information indicated that its use was for "disruptive behavior"' (e.g., hetero or self-aggressiveness). The control group (n=19) consisted of children and adolescents with normal psychomotor development, recruited from the local community. The study was approved by the Research Ethics Committee of UFMG and all participants and/or legal guardians gave their written informed consent before entering the study.

Patients and controls were interviewed using a structured questionnaire to collect demographic (age and sex of child and guardians) and clinical data. Parents of children with ASD responded to the inventory Social and Communication Disorders Checklist (SCDC)^16^ and parents of children with ADHD respond to the Swanson, Nolan, and Pelham scale – version IV (SNAP-IV).^17^ SCDC is a self-completion questionnaire with 12 questions that assesses the severity of behaviors related to autism in the last six months.^16^ SNAP-IV is a self-administered instrument that, in its version of 26 items, evaluates the severity of inattentive, hyperactive/impulsive, and opponent/defiant behavior.^17^

All subjects included in the study had their weight (kg) and height (m) measured at the time of interview. BMI was calculated using the formula: weight (expressed in kg) divided by height (expressed in meters) squared (kg/m^2^). Age and sex-specific BMI *z*-score and percentiles of patients and controls were calculated using CDC 2000 growth charts.^18^ Overweight was defined as a percentile ≥85; obesity as a percentile ≥95; and underweight as a percentile ≤5. For adults, overweight was defined as BMI between 25 and 30kg/m^2^ and obesity as BMI over 30kg/m^2^.

Subjects were also evaluated for abdominal circumference, which was also converted into percentiles according to CDC 2007–2010 graphics.^19^ Anthropometric measurements were taken with participants in light clothes and barefoot, using a portable digital scale, wall mounted stadiometer, and inelastic tape measure. Waist circumference was measured over the skin in the upper part of the lateral border of the right ilium and at the end of normal expiration, and the nearest millimeter was recorded.^20^

Statistical analyzes were performed using SPSS software version 22.0 (SPSS Inc., Chicago, IL, USA). Descriptive statistics were used to present the sociodemographic and clinical characteristics of the sample. Association between dichotomous variables was assessed with Pearson's chi-square or Fisher's exact test, as appropriate. Continuous variables were expressed as mean and standard deviation. All variables were tested for normal distribution using the Shapiro–Wilk test and no data had a normal distribution. Therefore, the differences between groups were compared using the Mann–Whitney test or Kruskal–Wallis test, as appropriate. Correlations were investigated using the Spearman coefficient. All *p*-values were two-tailed and the significance level of *p*<0.05 was used.

## Results

There was prevalence of male in the ASD (86.9%), ADHD (78.9%), and control (86.9%) groups. There was no statistically significant difference between groups (*p*=0.66). Also, there was no difference in age between individuals with ASD (8.4±4.2; range: 2–18 years), ADHD (8.5±2.4; range: 5–15 years), and controls (8.6±2.9; range: 5–15 years) (*p*=0.603).

Age and sex-specific BMI *z*-score and percentiles of controls were significantly lower than those of patients with ASD and ADHD (Table 1). Similar findings were seen when groups were compared regarding weight change proportions, with higher percentage of overweight and obesity in both groups of patients (ASD and ADHD) compared with the control group (Table 1). On the other hand, there was no significant difference between patients with ADHD and ASD in any of the comparisons (Table 1). Regarding waist circumference percentile, groups with ASD (56.4±29.9) and ADHD (65.0±18.3) also did not differ (*p*=0.453).

**Table 1:** Comparison of body mass index (BMI) and weight change between individuals with attention deficit/hyperactivity disorder (ADHD) and autism spectrum disorder (ASD) and controls.

		Controls (n=19)	ADHD (n=23)	ASD (n=69)	*p* -value
*BMI percentile* a		35.8±28.5	64.6±30.2	62.9±32.0	**0.006**
*BMI Z* - *score* a		−0.55±1.13	0.87±1.42	0.49±1.51	**0.002**
*Weight changes*					0.044
	Underweight	21.0%	0.00%	6.7%	
	Normal	73.7%	65.2%	53.3%	
	Overweight	0.00%	17.5%	18.3%	
	Obesity	5.3%	17.4%	21.7%	

a Values are expressed as mean±standard deviation.

BMI, body mass index. Bold indicates *p* -value significant at <0.05.

The severity of ADS symptoms did not correlate with anthropometric measurements (Table 2). In ADHD group, the BMI percentile correlated negatively with the severity of opposition and defiance symptoms of and there was no correlation with inattention or hyperactivity/impulsivity symptoms.

**Table 2:** Correlation of clinical features with body mass index (BMI) and waist circumference in individuals with autism spectrum disorder (ASD) and attention deficit/hyperactivity disorder (ADHD).

			BMI percentile	BMI *Z* -score	AC percentile
**ASD (n=69)**					
	*SCDC*				
		Correlation coefficient	0.059	0.062	−0.009
		Sig. (2 tailed)	0.727	0.717	0.960

**ADHD (n=23)**					
	*SNAP-I*				
		Correlation coefficient	−0.200	−0.251	−0.667
		Sig. (2 tailed)	0.555	0.457	0.219

	*SNAP-HI*				
		Correlation coefficient	−0.230	−0.331	0.803
		Sig. (2 tailed)	0.496	0.320	0.102

	*SNAP-OD*				
		Correlation coefficient	**−0.621**	−0.370	0.410
		Sig. (2 tailed)	**0.041**	0.263	0.493

AC, abdominal circumference; SCDC, Social and Communication Disorders Checklist; SNAP-I, SNAP-IV inattention subscale; SNAP-HI, SNAP-IV hyperactivity subscale; SNAP-OD, SNAP-IV opposition and defiance subscale. Bold indicates *p* -value significant at <0.05.

Of ADHD patients, 20 (87%) were taking psychiatric drugs (Table 3). Among ASD patients, 26 (37.7%) were taking psychiatric drugs. In ASD group, there was a tendency for a higher percentage of BMI (*p*=0.06) in participants taking risperidone. Of the 21 participants taking risperidone, 5 (23.8%) of guardians spontaneously reported seen a significant increase in appetite after drug initiation. Of the total 28 participants in use of methylphenidate, when faced with an open question on side effects, 13 (44.8%) guardians spontaneously reported that there was a significant decrease in appetite with the drug.

**Table 3:** Analysis of associations between drug use and body mass index (BMI) and waist circumference in individuals with autism spectrum disorder (ASD) and attention deficit/hyperactivity disorder (ADHD).

			BMI percentile (mean±SD)	BMI *Z* -score (mean±SD)	AC percentile (mean±SD)
**ASD (n=69)**					
	Risperidone				
		Yes (n=19)	72.6±26.8	1.0±1.3	63.7±27.7
		No (n=50)	58.4±33.6	0.2±1.5	47.5±31.0
		*p* -value	0.068	0.076	0.132

	*Methylphenidate*				
		Yes (n=9)	56.3±31.8	0.4±1.5	49.4±28.5
		No (n=60)	64.0±32.3	0.5±1.5	59.2±30.6
		*p* -value	0.627	0.641	0.333

	*Antidepressant*				
		Yes (n=65)	49.3±39.6	0.3±1.6	50.6±33.7
		No (n=4)	63.8±31.7	0.5±1.5	57.2±29.9
		*p* -value	0.501	0.483	0.712

**ADHD (n=23)**					
	Risperidone				
		Yes (n=2)	59.6±18.7	0.2±0.5	64.3±18.8
		No (n=21)	65.1±31.3	0.92±1.8	75.0±0.0
		*p* -value	0.640	0.569	0.625

	*Methylphenidate*				
		Yes (n=19)	63.6±30.2	0.8±1.5	95.0±0.0
		No (n=4)	74.4±32.7	1.1±1.4	63.0±17.1
		*p* -value	0.409	0.557	0.125

	*Antidepressant*				
		Yes (n=2)	70.3±33.8	0.7±1.2	75.0±0.0
		No (n=21)	64.1±30.7	0.9±1.5	63.6±19.3
		*p* -value	0.791	0.957	0.417

BMI, body mass index; AC, abdominal circumference.

Parents of participants with ASD and ADHD did not differ with respect to anthropometric measurements (Table 4). There was also no significant difference between parents of individuals with ASD and ADHD regarding frequency of overweight and obesity (*p*=0.34).

**Table 4:** Anthropometric data and frequency of weight change of parents of individuals with attention deficit/hyperactivity disorder (ADHD) and autism spectrum disorder (ASD).

		Parents of individuals with ASD (n=69)	Parents of individuals with ADHD (n=23)	*p* -value
*Weight (kg)* a		70.6±22.8	65.1±12.2	0.703
*Height (m)* a		1.6±0.1	1.6±0.1	0.241
*BMI (kg/m* ^*2*^ *)* a		26.8±6.9	25.8±4.0	0.979
*Abdominal circumference (cm)* a		84.5±13.1	86.3±10.6	0.273

*Weight change*				
	Normal BMI	37 (53.6%)	13 (56.5%)	0.336
	Overweight	17 (24.6%)	8 (34.8%)	
	Obesity	15 (21.7%)	2 (8.7%)	

a Values are expressed as mean±standard deviation.

BMI, body mass index.

## Discussion

Overweight and obesity are public health problems in the general population. The incidence of many chronic diseases in adulthood is directly related to childhood obesity.^1^ Obesity increases the risk of children having short and long term health problems, such as diabetes, cardiovascular and psychosocial diseases. International studies indicate that children and adolescents with ASD and ADHD may be particularly vulnerable to such weight changes.^4^^–^ 13 This work shows that Brazilian children and adolescents with ASD and ADHD also appear to be more prone to overweight and obesity compared to the general population.

Few Brazilian studies have investigated this issue. Domingues evaluated 30 children in a special school of Campo Grande and found that four (13.3%) were obese and seven (23.3%) were underweight.^21^ Emidio et al. evaluated 23 autistic children and adolescents and found that three (13%) were underweight, five (21.7%) were overweight, and six (26.1%) were obse.^22^ Although both studies indicate high rates of overweight and obesity in children and adolescents with ASD, the absence of a control group significantly limited the interpretation of the findings.

Nascimento et al. found no difference in frequency of overweight among children and adolescents with “ADHD-indicative” and controls, or when comparing the BMI of both the groups.^23^ A limitation of this study is the inclusion of children with only “ADHD-indicative”, without a confirmed diagnosis of the disorder. Moreover, the study did not properly set the variable “overweight”, as well as directly compared the BMI of the two groups, instead of comparing the adjusted percentile for age. In contrast, Paranhos et al. reported that all 70 children and adolescents with ADHD evaluated at the University Hospital of Brasilia were eutrophic.^24^ However, this study also did not adequately defined the weight change and did not use the percentile variable and/or the age and sex-specific BMI *z*-scores.

There are few published studies that compared BMI and frequency of overweight/obesity between developmental disorders. Curtin et al. performed a review of medical records of children and adolescents in care in a tertiary center for neuropsychiatric disorders and found a frequency of overweight and obesity of, respectively, 29% and 17.3% in ADHD patients and 35.7% and 19% in ASD patients.^25^ The Curtin study limitation is that anthropometric measurements were obtained from medical records, there was no standardization in the form of data collection. Moreover, there was no control group or comparisons between groups. Chen et al. evaluated only the frequency of obesity (BMI ≥95th percentile) in children and adolescents with chronic desases (physical, developmental, and behavioral) and found frequency of 23.4% in ASD patients and 18.9% in ADHD patients.^26^ The study by Chen et al. also has a number of limitations, such as using data from the National Survey of Children's Health (NSCH 2003), in which all information was collected by telephone; the possibility of having the same participant with comorbidities including ASD and ADHD at the same time; and no statistical calculation for comparison between groups. More recently, Philips et al. used data from the National Health Interview Survey (2008–2010) to compare the frequency of underweight, overweight, and obesity in adolescents with various developmental disorders.^13^ Obesity and underweight were more common in all neurodevelopmental disorders, but the frequency of obesity was particularly high in the subgroup with ASD (31.8%), whereas patients with ADHD had a lower prevalence (17.6%). The frequency of overweight was similar between the groups with developmental disorders (ASD: 20.9%; ADHD: 18.0%) and children with typical development (18.2%). The study should also be interpreted with caution because of its limitations, including the medical and anthropometric data collection only by interviewing the guardians; non-pairing by sex between the control and ASD and ADHD groups, there was prevalence of female adolescents in the control group^13^; and the high frequency of comorbidities between ASD and ADHD (60% of ASD patients also had ADHD).

A common limitation of many studies of weight changes in people with ASD and ADHD is that the authors use only the BMI as base. Such an approach does not allow the direct measurement of body composition and/or distribution of body fat, there may be increased BMI because of increased muscle mass. Waist circumference measurement is easy to obtain and can indicate the presence of visceral adiposity in children.^27^ In this context, the present study showed that groups with ASD and ADHD did not differ from each other or in relation to BMI (high compared to control) nor in relation to waist circumference.

The severity of ASD and ADHD symptoms did not correlate with weight change. However, opposition and defiance symptoms negatively correlated with BMI percentile in ADHD participants. Korczak et al. noted that ADHD could predict obesity later in life, but according to the authors this association would be completely explained by the presence of behavior disorder in childhood.^28^ Although opposition-defiance and behavior symptoms are often correlated, this seemingly discrepant finding raises the question of whether these conditions may have differential effect on feeding behavior (e.g., refusal to eat and frequent tantrums related to food in the opponent-defiant disorder).

Consistent with the medical literature, there was a trend towards the association of risperidone intake and higher BMI percentile in ASD patients. Increased appetite and metabolic alterations are common side effects of risperidone.^20^ Interestingly, although the parents of children taking methylphenidate have reported decreased appetite with this substance, this was not reflected in lower BMI in ADHD children. It was not possible to compare the BMI of ADHD children taking or not taking methylphenidate because almost all of them were taking this drug. However, although methylphenidate common side effect is decreased appetite, other studies have found no difference in BMI between ADHD children receiving or not methylphenidate.^6^^,^ 7

Finally, it was seen for the first time in the literature that parents of children with ASD and ADHD did not differ in BMI, waist circumference, and frequency of overweight/obesity. Although nearly half of parents are overweight/obese, unfortunately it was not possible to compare them with the control group because the children and adolescents of the control group were examined at school without the presence of their parents. Some studies suggest that obesity in parents is a risk factor for ASD and ADHD. However, more investigations are needed to assess factors underlying this possible association, such as genetic/epigenetic influence, family habits, inflammatory changes resulting from obesity during gametogenesis and pregnancy, among others.^14^^,^ 29

These results should be interpreted with caution. Due to its cross-cutting nature, one cannot infer causality between ASD, ADHD, and higher frequency of overweight and obesity. Furthermore, this study included a relatively small convenience sample of ADHD patients and controls. All patients were recruited from a single tertiary center where more complex cases with more pronounced symptoms of ADHD and ASD are usually treated, which could compromise the external validity of the study. Moreover, there were a significant proportion of underweight children in the control group, which highlighted a peculiarity of the sample involved in this work. The lack of waist circumference measurement in the controls and anthropometric measurement in their parents is also a limitation that should be mentioned. On the other hand, consecutive sampling, direct and careful measurement of anthropometric parameters of patients and community controls without developmental disorders, diagnosis and evaluation of patients by experienced clinicians, sample matched by gender and age, and the exclusion of comorbid cases of ADHD and ASD are strengths of the study that should be highlighted.

In summary, this study showed that children and adolescents with ASD and ADHD followed-up in outpatient clinics are at increased risk of overweight and obesity than their typically developing peers. Drugs such as risperidone may have some causal role and patients taking these substances require close monitoring for metabolic changes. However, genetic and environmental factors (e.g., poor eating habits and sedentary lifestyle) should also be considered. We emphasize that obesity and overweight are general risk factors for disorders and responsible for worsening the quality of life. Thus, it is very important that physicians routinely assess the weight of their patients with ASD and ADHD and give them advises on healthy living habits.
