# Supplementary material for: Frequency of overweight and obesity in children and adolescents with autism and attention deficit/hyperactivity disorder
Source: Rev Paul Pediatr. 2016 Jan-Mar;34(1):71–7. doi: 10.1016/j.rppede.2015.12.006 (PMC4795724; doi:10.1016/j.rppede.2015.12.006)
Supplement: Translation

# Translation: Frequência de sobrepeso e obesidade em crianças e adolescentes com autismo e transtorno do déficit de atenção/hiperatividade

**Authors:** Arthur Kummer, Izabela Guimarães Barbosa, David Henrique Rodrigues, Natália Pessoa Rocha, Marianna da Silva Rafael, Larissa Pfeilsticker, Ana Cristina Simões e Silva, Antônio Lúcio Teixeira

**Affiliations:** a Faculdade de Medicina, Universidade Federal de Minas Gerais (UFMG), Belo Horizonte, MG, Brasil; b Universidade Federal de Juiz de Fora (UFJF), Governador Valadares, MG, Brasil

**Keywords:** Transtorno do déficit de atenção e hiperatividade, Transtorno autístico, Obesidade pediátrica, Sobrepeso

## Abstract

**Objetivo:**

Avaliar a frequência de sobrepeso e obesidade em crianças e adolescentes com transtorno do espectro do autismo (TEA) e transtorno do déficit de atenção/hiperatividade (TDAH) e em seus pais, em comparação com crianças e adolescentes da comunidade sem transtornos do desenvolvimento.

**Métodos:**

Medidas antropométricas foram coletadas de 69 pacientes com TEA (8,4±4,2 anos), 23 com TDAH (8,5±2,4) e 19 controles sem transtornos desenvolvimentais (8,6±2,9) entre agosto e novembro de 2014. Os pais dos pacientes com TEA e TDAH também foram avaliados em relação aos parâmetros antropométricos. Sobrepeso foi definido como percentil ≥85; obesidade como percentil ≥95; e baixo peso como percentil ≤5. Para os adultos, sobrepeso foi definido como IMC entre 25 e 30kg/m^2^ e obesidade, IMC acima de 30kg/m^2^.

**Resultados:**

Crianças e adolescentes com TEA e TDAH exibiram maior percentil (*p<*0,01) e escore-z (*p<*0,01) do IMC em relação aos controles, bem como frequência mais elevada de sobrepeso e obesidade (*p=*0,04). Os pacientes com TEA e TDHA não diferiram entre si quanto a essas variáveis ou quanto à circunferência abdominal. Os pais das crianças com TEA e TDAH também não diferiram entre si.

**Conclusões:**

Crianças e adolescentes com TEA e TDAH estão em maior risco de ter sobrepeso e obesidade em relação a crianças da comunidade sem problemas do desenvolvimento.

## Introdução

A prevalência de sobrepeso e obesidade em países desenvolvidos é alarmante, atinge 31,8% das crianças e adolescentes.^1^ Nos EUA, apesar dos contínuos esforços para reduzir esses problemas de saúde pública, as taxas têm se mantido estáveis na última década.^1^ No Brasil, estudos epidemiológicos têm apontado uma frequência crescente de sobrepeso e obesidade nessa faixa etária.^2^^,^ 3 A comparação entre a Pesquisa Nacional sobre Saúde e Nutrição/PNSN de 1989 e a Pesquisa de Orçamentos Familiares feita entre 2008-2009 mostra que a frequência de sobrepeso em crianças entre cinco e nove anos aumentou de aproximadamente 9% para 33%.^3^

Estudos internacionais observam associação entre sobrepeso/obesidade infantil e transtornos psiquiátricos como o transtorno do espectro autista (TEA) e o transtorno do déficit de atenção/hiperatividade (TDAH). Apesar disso, não está claro se essa associação é própria do TEA e do TDAH ou comum a problemas comportamentais e desenvolvimentais de forma geral. Além disso, é possível que a associação seja bidirecional, ou seja, não apenas problemas comportamentais podem levar à obesidade, mas a obesidade pode ser um fator de risco para o desenvolvimento de problemas comportamentais e desenvolvimentais.^4^

A maioria dos estudos sobre o estado nutricional de jovens e adultos com TDAH aponta para uma frequência elevada de sobrepeso e obesidade, bem como um índice de massa corporal (IMC) médio maior no grupo com TDAH em relação a controles sem transtornos desenvolvimentais.^5^^-^ 7 A frequência de obesidade é maior em adultos com TDAH do que em adultos com história de TDAH na infância, mas cujos sintomas remitiram na vida adulta.^8^ Da mesma forma, jovens com obesidade também têm maior frequência de TDAH.^5^ Além disso, problemas comportamentais, como o TDAH, dificultam o tratamento da obesidade.^9^

De modo similar, estudos também indicam que crianças e adolescentes com TEA têm sobrepeso e obesidade em maior frequência.^10^^-^ 13 No TEA, as alterações ponderais têm sido associadas a alterações do sono,^10^^,^ 11 maior idade^11^ e uso de alimentos como recompensa,^12^ entre outros. Além disso, pais e mães de crianças com autismo também têm maior frequência de obesidade.^14^ Tais fatores sugerem uma complexa interação entre fatores genéticos, moleculares e comportamentais.

Apesar desses dados alarmantes, não há estudos sobre o estado nutricional de crianças com TEA e TDAH no Brasil. Além disso, poucos estudos compararam o IMC e/ou a frequência de sobrepeso entre diferentes transtornos do desenvolvimento.

Nesse contexto, o objetivo do presente trabalho foi avaliar a frequência de sobrepeso e obesidade em crianças e adolescentes com TEA e TDAH e seus pais.

## Método

Crianças e adolescentes atendidos no Ambulatório de TEA (n=69) e no Ambulatório de Déficit de Atenção (n=23) do Serviço de Psiquiatria do Hospital das Clínicas da Universidade Federal de Minas Gerais (UFMG), Brasil, e seus responsáveis foram convidados a participar deste estudo. Os dados foram coletados entre agosto e novembro de 2014 e não houve recusa para participar de nenhuma das pessoas convidadas. Todos os pacientes com TEA e TDAH preencheram os critérios de diagnóstico do DSM-5^15^. Esse foi considerado o padrão-ouro. Pacientes do grupo de TEA não tinham TDAH como comorbidade e vice-versa. De nota, alguns pacientes com TEA estavam em uso de metilfenidato, mas informações de prontuário indicavam que seu uso fora para “comportamentos disruptivos” (ex.: hetero ou autoagressividade). O grupo controle (n=19) foi composto por crianças e adolescentes com desenvolvimento neuropsicomotor normal, recrutados na comunidade local. O estudo foi aprovado pelo Comitê de Ética em Pesquisa da UFMG e todos os participantes e/ou responsáveis legais pelas crianças assinaram o termo de consentimento livre e esclarecido antes da entrada no estudo.

Os pacientes e controles foram entrevistados por meio de questionário estruturado para a coleta de dados demográficos (idade da criança e do responsável, sexo) e clínicos. Os pais das crianças com TEA responderam ao inventário Social and Communication Disorders Checklist (SCDC)^16^ e os pais das crianças com TDAH responderam à escala de Swanson, Nolan e Pelham – versão IV (SNAP-IV)^17^. O SCDC é um questionário autopreenchível com 12 perguntas, que avalia a gravidade de comportamentos relacionados ao autismo nos últimos seis meses.^16^ O SNAP-IV é um instrumento autopreenchível que, na sua versão de 26 itens, avalia a gravidade do comportamento desatento, hiperativo/impulsivo e opositor/desafiador.^17^

Todos os sujeitos incluídos na pesquisa tiveram seu peso (kg) e estatura (m) mensurados no momento da entrevista. O IMC foi calculado por meio da fórmula: peso (expresso em Kg) dividido pela estatura (expressa em m) elevada ao quadrado (kg/m^2^). Os percentis e escores-z do IMC específicos para a idade e gênero de pacientes e controles foram calculados a partir dos gráficos de crescimento do CDC 2000.^18^ Sobrepeso foi definido como percentil ≥85; obesidade como percentil ≥95; e baixo peso como percentil ≤5. Para os adultos, sobrepeso foi definido como IMC entre 25 e 30 kg/m^2^ e obesidade, IMC acima de 30 kg/m^2^.

Os sujeitos foram ainda avaliados quanto à circunferência abdominal, a qual também foi convertida em percentis de acordo com os gráficos do CDC 2007-2010.^19^ As medidas antropométricas foram tomadas com os participantes em roupas leves e descalços, por meio de balança digital portátil, estadiômetro montado na parede e fita métrica não elástica. A circunferência abdominal foi medida sobre a pele, na parte mais alta da borda lateral do íleo direito e no fim da expiração normal e registrou-se o milímetro mais próximo.^20^

As análises estatísticas foram feitas com o *software* SPSS versão 22.0 (SPSS Inc., Chicago, IL, EUA). Estatística descritiva foi usada para apresentar as características sociodemográficas e clínicas da amostra. Associação entre variáveis dicotômicas foi avaliada com o teste do qui-quadrado de Pearson ou o teste exato de Fisher, quando apropriado. Variáveis contínuas foram expressas como média e desvio padrão. Todas as variáveis foram testadas quanto à normalidade de distribuição por meio do teste de Shapiro-Wilk e nenhum dado apresentou distribuição normal. Portanto, as diferenças entre os grupos foram comparadas com o teste de Mann-Whitney ou Kruskal-Wallis, quando apropriado. Correlações foram averiguadas por meio do coeficiente de Spearman. Todos os valores de *p* foram bicaudais e o nível de significância de *p<*0,05 foi usado.

## Resultados

Gênero masculino foi a maioria no grupo com TEA (86,9%), TDAH (78,9%) e Controle (86,9%). Não houve diferença estatisticamente significativa entre os grupos (*p=*0,66). Também não ouve diferença de idade entre indivíduos com TEA (8,4±4,2; variação: 2-18 anos), TDAH (8,5±2,4; variação: 5-15 anos) e controles (8,6±2,9; variação: 5-15 anos) (*p=*0,603).

O percentil do IMC e o escore-z específicos para sexo e idade dos controles foram significativamente mais baixos do que os dos pacientes com TEA e TDAH (tabela 1). Achados semelhantes se reproduziram quando os grupos foram comparados em relação às proporções de alterações de peso e observou-se maior percentual de sobrepeso e obesidade nos dois grupos de pacientes (TEA e TDHA) em comparação com o grupo controle (tabela 1). Por outro lado, não houve diferença significativa entre pacientes com TDAH e TEA em nenhuma das comparações (tabela 1). Com relação ao percentil da circunferência abdominal, os grupos com TEA (56,4±29,9) e TDAH (65,0±18,3) também não diferiram entre si (*p=*0,453).

**Tabela 1:** Comparação de índice de massa corporal (IMC) e alterações ponderais entre indivíduos com transtorno de déficit de atenção/hiperatividade (TDAH), transtorno do espectro do autismo (TEA) e controles

		Controles (n *=* 19)	TDAH (n *=* 23)	TEA (n *=* 69)	*p* ‐valor
*Percentil do IMC* a		35,8±28,5	64,6±30,2	62,9±32,0	**0,006**
*Escore‐z do IMC* a		−0,55±1,13	0,87±1,42	0,49±1,51	**0,002**
*Alterações ponderais*					0,044
	Baixo peso	21,0%	0,00%	6,7%	
	Normal	73,7%	65,2%	53,3%	
	Sobrepeso	0,00%	17,5%	18,3%	
	Obesidade	5,3%	17,4%	21,7%	

a Valores expresso em média ± desvio padrão.

IMC, índice de massa corpórea. Os negritos indicam *p* ‐valor <0,05.

A gravidade dos sintomas de TEA não se correlacionou com as medidas antropométricas (tabela 2). No grupo de TDAH, o percentil do IMC se correlacionou negativamente com a gravidade de sintomas de oposição e desafio e não houve correlação com sintomas de desatenção ou hiperatividade/impulsividade.

**Tabela 2:** Correlações de características clínicas com índice de massa corporal (IMC) e circunferência abdominal em indivíduos com transtorno do espectro do autismo (TEA) e transtorno de déficit de atenção/hiperatividade (TDAH)

			Percentil de IMC	Escore‐z de IMC	Percentil de CA
**TEA (n = 69)**					
	*SCDC*				
		Coeficiente de correlação	0,059	0,062	−0,009
		Sig. (2 extremidades)	0,727	0,717	0,960

**TDAH (n = 23)**					
	*SNAP‐D*				
		Coeficiente de correlação	−0,200	−0,251	−0,667
		Sig. (2 extremidades)	0,555	0,457	0,219

	*SNAP‐HI*				
		Coeficiente de correlação	−0,230	−0,331	0,803
		Sig. (2 extremidades)	0,496	0,320	0,102

	*SNAP‐OD*				
		Coeficiente de correlação	**−0,621**	−0,370	0,410
		Sig. (2 extremidades)	**0,041**	0,263	0,493

CA, circunferência abdominal; SCDC, Social and Communication Disorders Checklist; SNAP‐D, Subescala de desatenção do SNAP‐IV; SNAP‐HI, Subescala de hiperatividade do SNAP‐IV; SNAP‐OD, Subescala de oposição e desafio do SNAP‐IV.

Dos pacientes com TDAH, 20 (87%) estavam em uso de psicofármacos (tabela 3). Entre os pacientes com TEA, 26 (37,7%) estavam em uso de psicofármacos. No grupo de TEA, houve tendência de os participantes em uso de risperidona apresentarem percentil mais elevado do IMC (*p=*0,06). Dos 21 participantes em uso de risperidona, 5 (23,8%) dos responsáveis relataram espontaneamente terem observado aumento significativo do apetite após início do fármaco. Do total de 28 participantes em uso de metilfenidato, frente a uma pergunta aberta sobre efeitos colaterais, 13 (44,8%) responsáveis relataram espontaneamente que houve diminuição significativa do apetite com o fármaco.

**Tabela 3:** Análise de associações entre uso de medicamento com índice de massa corporal (IMC) e circunferência abdominal em indivíduos com transtorno do espectro do autismo (TEA) e transtorno de déficit de atenção/hiperatividade (TDAH)

			Percentil de IMC (média±DP)	Escore‐z de IMC (média±DP)	Percentil de CA (média±DP)
**TEA (n = 69)**					
	*Risperidona*				
		Sim (n *=* 19)	72,6±26,8	1,0±1,3	63,7±27,7
		Não (n *=* 50)	58,4±33,6	0,2±1,5	47,5±31,0
		*p* ‐valor	0,068	0,076	0,132

	*Metilfenidato*				
		Sim (n *=* 9)	56,3±31,8	0,4±1,5	49,4±28,5
		Não (n *=* 60)	64,0±32,3	0,5±1,5	59,2±30,6
		*p* ‐valor	0,627	0,641	0,333

	*Antidepressivo*				
		Sim (n *=* 65)	49,3±39,6	0,3±1,6	50,6±33,7
		Não (n *=* 4)	63,8±31,7	0,5±1,5	57,2±29,9
		*p* ‐valor	0,501	0,483	0,712

**TDAH (n=23)**					
	*Risperidona*				
		Sim (n *=* 2)	59,6±18,7	0,2±0,5	64,3±18,8
		Não (n *=* 21)	65,1±31,3	0,92±1,8	75,0±0,0
		*p* ‐valor	0,640	0,569	0,625

	*Metilfenidato*				
		Sim (n *=* 19)	63,6±30,2	0,8±1,5	95,0±0,0
		Não (n *=* 4)	74,4±32,7	1,1±1,4	63,0±17,1
		*p* ‐valor	0,409	0,557	0,125

	*Antidepressivo*				
		Sim (n *=* 2)	70,3±33,8	0,7±1,2	75,0±0,0
		Não (n *=* 21)	64,1±30,7	0,9±1,5	63,6±19,3
		*p* ‐valor	0,791	0,957	0,417

IMC, índice de massa corpórea; CA, circunferência abdominal.

Os pais dos participantes com TEA e TDAH não diferiram estatisticamente com relação às medidas antropométricas (tabela 4). Também não houve diferença significativa entre pais de indivíduos com TEA e TDAH quanto à frequência de sobrepeso e obesidade (*p=*0,34).

**Tabela 4:** Dados antropométricos e frequência de alterações ponderais dos pais dos indivíduos com transtorno de déficit de atenção/hiperatividade (TDAH) e transtorno do espectro do autismo (TEA)

		Pais de indivíduos com TEA (n *=* 69)	Pais de indivíduos com TDAH (n *=* 23)	*p* ‐valor
*Peso (kg)* a		70,6±22,8	65,1±12,2	0,703
*Altura (m)* a		1,6±0,1	1,6±0,1	0,241
*IMC (kg/m* ^*2*^ *)* a		26,8±6,9	25,8±4,0	0,979
*Circunferência abdominal (cm)* a		84,5±13,1	86,3±10,6	0,273

*Alterações ponderais*				
	IMC normal	37 (53,6%)	13 (56,5%)	0,336
	Sobrepeso	17 (24,6%)	8 (34,8%)	
	Obesidade	15 (21,7%)	2 (8,7%)	

a Valores expresso em média±desvio padrão.

IMC, índice de massa corpórea.

## Discussão

O sobrepeso e a obesidade são problemas de saúde pública na população geral. A incidência de muitas doenças crônicas na vida adulta está diretamente ligada à obesidade na infância.^1^ A obesidade aumenta o risco na criança de problemas em curto e longo prazo, como diabete, doenças cardiovasculares e psicossociais. Estudos internacionais indicam que crianças e adolescentes com TEA e TDAH podem ser particularmente vulneráveis a essas alterações ponderais.^4^^-^ 13 Este trabalho mostra que as crianças e adolescentes brasileiros com TEA e TDAH também parecem ser mais propensos a sobrepeso e obesidade, quando comparados com a população geral.

Poucos trabalhos nacionais investigaram essa questão. Domingues avaliou 30 crianças de uma escola especial de Campo Grande e observou que quatro (13,3%) estavam obesas e sete (23,3%) com baixo peso.^21^ Emidio *et al*. avaliaram 23 crianças e adolescentes autistas e observaram que três (13%) apresentavam baixo peso, cinco (21,7%) tinham sobrepeso e seis (26,1%) exibiam obesidade.^22^ Apesar de ambos os trabalhos indicarem frequência elevada de sobrepeso e obesidade em crianças e adolescentes com TEA, a ausência de grupo controle limitou significativamente a interpretação dos achados.

Nascimento *et al*. não encontraram diferença na frequência de sobrepeso entre crianças e adolescentes com “indicativo de TDAH” e controles, nem ao comparar o IMC dos dois grupos.^23^ Uma limitação desse estudo é a inclusão de crianças apenas com “indicativo de TDAH”, sem diagnóstico do transtorno confirmado. Além disso, o estudo não definiu adequadamente a variável “sobrepeso”, bem como comparou diretamente o IMC dos dois grupos, em vez de comparar o percentil ajustado para a idade. Em contrapartida, Paranhos *et al*. relataram que todas as 70 crianças e adolescentes com TDAH avaliados no Hospital Universitário de Brasília eram eutróficos.^24^ Porém, esse estudo também não definiu adequadamente as alterações ponderais e não usou a variável de percentil e/ou o escore-z do IMC específico para sexo e idade.

Há poucos estudos na literatura internacional que compararam transtornos do desenvolvimento entre si quanto ao IMC e à frequência de sobrepeso/obesidade. Curtin *et al*. fizeram revisão de prontuário de crianças e adolescentes em atendimento em centro terciário para transtornos neuropsiquiátricos e encontraram uma frequência de sobrepeso e de obesidade de, respectivamente, 29% e 17,3% em pacientes com TDAH e de 35,7% e 19% em pacientes com TEA.^25^ O estudo de Curtin *et al*. tem a limitação de que as medidas antropométricas foram obtidas no prontuário, não houve padronização na forma de obtenção dos dados. Além disso, não havia grupo controle e não foram feitas comparações entre os grupos. Chen *et al*. avaliaram apenas a frequência de obesidade (percentil de IMC ≥95) em crianças e adolescentes com doenças crônicas (físicas, desenvolvimentais e comportamentais) e encontraram frequência de 23,4% nos pacientes com TEA e 18,9% naqueles com TDAH.^26^ O estudo de Chen *et al*. também conta com uma série de limitações, como o uso de dados provenientes do National Survey of Children's Health (NSCH 2003), em que todas as informações foram coletadas por telefone; a possibilidade de o mesmo participante ter comorbidades, incluindo TEA e TDAH ao mesmo tempo; e não ter feito cálculo estatístico para comparar os grupos entre si. Mais recentemente, Philips *et al*. usaram dados do National Health Interview Survey (2008-2010) para comparar a frequência de baixo peso, sobrepeso e obesidade em adolescentes com vários transtornos do desenvolvimento^13^ Obesidade e baixo peso foram mais comuns em todos os transtornos neurodesenvolvimentais, mas a frequência de obesidade foi particularmente elevada no subgrupo com TEA (31,8%), enquanto os pacientes com TDAH tiveram prevalência mais baixa (17,6%). A frequência de sobrepeso foi similar entre os grupos com transtornos do desenvolvimento (TEA: 20,9%; TDAH: 18,0%) e as crianças com desenvolvimento típico (18,2%). O estudo também deve ser interpretado com cautela em virtude de suas limitações, que incluem a coleta de dados médicos e antropométricos apenas por entrevista com o responsável; o não pareamento por gênero entre grupo controle e os grupos de TEA e TDAH, houve maior proporção de adolescentes do gênero feminino no grupo controle^13^; e a elevada frequência de comorbidades entre TEA e TDAH (60% dos pacientes com TEA também tinham TDAH).

Uma limitação comum aos diversos estudos sobre alterações ponderais em pessoas com TEA e TDAH é que os autores tomam por base apenas o IMC. Tal abordagem não permite aferir diretamente a composição corporal e/ou a distribuição da gordura corporal, pode haver elevação do IMC em virtude de maior massa muscular. A aferição da circunferência abdominal é de fácil obtenção e capaz de indicar a presença de adiposidade visceral na população pediátrica.^27^ Nesse contexto, o presente estudo mostrou que os grupos com TEA e com TDAH não diferem entre si nem em relação ao IMC (elevado em relação ao controle) nem em relação à circunferência abdominal.

A gravidade dos sintomas de TEA e TDAH não se correlacionou com as alterações ponderais. Porém, os sintomas de oposição e desafio se correlacionaram negativamente com o percentil do IMC nos participantes com TDAH. Korczak *et al*. haviam observado que TDAH poderia predizer obesidade na vida adulta, mas, segundo os autores, tal associação seria completamente explicada pela presença de transtorno de conduta na infância.^28^ Apesar de sintomas de oposição-desafio e de conduta se correlacionarem frequentemente, esse achado aparentemente discrepante levanta a questão de se esses quadros podem exercer efeito diferencial sobre o comportamento alimentar (por exemplo, com recusa alimentar e birras frequentes relacionadas à comida no transtorno opositor-desafiador).

Condizente com a literatura médica, houve tendência de o uso de risperidona se associar com percentil mais alto do IMC nos pacientes com TEA. Aumento do apetite e alterações metabólicas são efeitos colaterais comuns da risperidona.^20^ Curiosamente, apesar de os pais de crianças em uso de metilfenidato relatarem diminuição do apetite com essa substância, isso não se refletiu em menor IMC nas crianças com TDAH. Não foi possível comparar o IMC de crianças com TDAH com ou sem uso de metilfenidato em virtude de quase todos estarem em uso desse fármaco. No entanto, apesar de o metilfenidato ter como efeito colateral comum a diminuição do apetite, outros estudos não observaram diferença no IMC entre crianças com TDAH em uso ou não de metilfenidato.^6^^,^ 7

Por fim, observou-se, pela primeira vez na literatura, que pais das crianças com TEA e TDAH não diferem em relação ao IMC, circunferência abdominal e frequência de sobrepeso/obesidade. Apesar de quase metade dos pais estar com sobrepeso/obesidade, infelizmente não foi possível sua comparação com o grupo controle em virtude de as crianças e adolescentes do grupo controle terem sido examinados na escola, sem a presença de seus pais. Alguns estudos sugerem que a obesidade em pais e em mães são fatores de risco para TEA e TDAH. No entanto, são necessárias novas pesquisas para avaliar fatores subjacentes a essa possível associação, tais como influência genética/epigenética, hábitos familiares, alterações inflamatórias decorrentes da obesidade durante gametogênese e gestação, entre outros.^14^^,^ 29

Os resultados deste trabalho devem ser interpretados com cautela. Em razão de sua natureza transversal, não se pode inferir causalidade entre TEA, TDAH e a maior frequência de sobrepeso e obesidade. Além disso, este estudo incluiu uma amostra de conveniência relativamente pequena de portadores de TDAH e de controles. Todos os pacientes foram recrutados em um único centro terciário, onde casos mais complexos e com sintomas mais pronunciados de TDAH e TEA são normalmente atendidos, o que pode comprometer a validade externa do estudo. Além disso, houve, no grupo controle, uma proporção significativa de crianças com baixo peso, que ressaltou uma particularidade da amostra envolvida neste trabalho. A falta da medida de circunferência abdominal nos controles e das medidas antropométricas de seus pais também é uma limitação que devem ser mencionada. Por outro lado, a amostragem consecutiva, a aferição direta e criteriosa dos parâmetros antropométricos dos pacientes e controles da comunidade sem transtornos do desenvolvimento, o diagnóstico e avaliação dos pacientes por clínicos experientes, o pareamento da amostra por gênero e idade e a não inclusão de casos comórbidos de TDAH e TEA são pontos fortes do estudo que devem ser ressaltados.

Em resumo, o presente estudo mostrou que crianças e adolescentes acompanhados em ambulatório com TEA e TDAH apresentam maior risco de sobrepeso e obesidade do que seus pares com desenvolvimento típico. Fármacos como a risperidona podem exercer algum papel causal e os pacientes em uso dessas substâncias necessitam de monitoramento próximo quanto a alterações metabólicas. No entanto, fatores genéticos e ambientais (ex: maus hábitos alimentares e sedentarismo) também devem ser considerados. Ressalta-se que obesidade e sobrepeso são fatores de risco à saúde em geral e responsáveis por pioria da qualidade de vida. Dessa forma, torna-se de grande importância que clínicos façam rotineiramente avaliação ponderal e orientem seus pacientes com TEA e TDAH sobre hábitos saudáveis de vida.
